# International breast density and cancer risk assessment workshop: advances, challenges, and emerging solutions, a summary from the 11th IBDW, Kaua‘i 2025

**DOI:** 10.1186/s13058-026-02323-7

**Published:** 2026-09-10

**Authors:** John Shepherd, Kimberly Bertrand, Fredrik Strand, Celine Vachon, Karla Kerlikowske

**Affiliations:** 1 Population Science Program, University of Hawai’i Cancer Center, Honolulu, HI USA; 2 Boston University School of Medicine, MA Boston, USA; 3 Karolinska Institutet, Stockholm, Sweden; 4 Mayo Clinic, MN Rochester, USA; 5 Department of Medicine, University of California San Francisco, CA San Francisco, US

**Keywords:** Mammography, Radiomics, Deep learning, Background parenchymal enhancement, Polygenic risk score, Early-life exposures, Supplemental screening, Breast density notification, Masking, Chemoprevention

## Abstract

Breast density is one of the strongest risk factors for breast cancer and a key determinant of mammographic sensitivity, yet fundamental questions remain regarding its measurement, biology, and role in individualized risk management. The International Breast Density Workshop (IBDW) has convened biennially since 2002 to advance this field. The 11th IBDW was held in Līhue, Kaua’i, Hawai’i in June 2025, bringing together 85 unique abstracts from researchers, clinicians, and advocates representing 14 countries. The workshop was organized around five thematic sessions covering: the state of the art in risk assessment and imaging biomarkers; biological and environmental modulators of breast density; innovative technology and methods for imaging-based risk assessment; frontiers in integrating clinical, omic, and imaging biomarkers; and community engagement and health equity in screening strategies. An innovative Breast Cancer Risk Board, modeled on the clinical tumor board format, provided a forum for real-time audience engagement on individualized risk management cases. Across sessions, recurring themes included the rapid maturation of AI-based imaging biomarkers, the critical need for validation in racially and ethnically diverse populations, and the importance of standardized density measures for equitable screening recommendations. The 12th IBDW will be held in Maui, Hawai’i, June 2–4, 2027.

## Introduction

Other than age, high breast density is the strongest common predictor of who will develop breast cancer. Women with the highest quartile of dense breast volume are 4 to 6 times more likely to develop breast cancer than those in the first quartile [1, 2]. Further, the sensitivity of mammography is markedly reduced in dense breasts, leading to false negative exams [3]. Awareness around this so-called “masking effect” has led to notification laws at state and national levels, including the U.S [4, 5]., Canada [6], and Australia [7] to report breast density back to primary care physicians as well as patients. Even though the risk and masking effects of high breast density have been known and studied for over 30 years, less is known regarding the most effective way of measuring breast density, its confounders, its underlying biology, and its actual mechanism of risk.

In recent years, other breast imaging biomarkers and risk predictors have been studied and found to be independent risk factors for breast cancer, most recently breast image features identified using artificial intelligence. Thus, there is a current and increasing unmet need to understand the utility of novel imaging biomarkers in risk assessment and the underlying biological mechanisms by which they increase risk. The International Breast Density Workshop (IBDW) was established to address this unmet need. Starting in 2002, the IBDW has convened approximately every other year to bring together researchers, clinicians, advocates, and other interested parties to report on the most recent advances in this field.

The 2025 International Breast Density and Cancer Risk Assessment Workshop convened in Kaua‘i to explore the latest advances in breast density science, imaging technologies, risk modeling, and community engagement. The workshop featured 85 unique abstracts, including 8 invited plenary presentations and 77 poster abstracts, of which 17 were selected for oral presentation These abstracts represented the contributions of early-career and established researchers from 14 countries, reflecting the global scope and collaborative nature of the workshop. Countries represented included the United States, Sweden, Australia, Canada, the United Kingdom, Germany, the Netherlands, France, Norway, Italy, South Korea, Japan, China, and New Zealand.

The workshop was divided into five thematic sessions including (1) *State of the Art in Risk Assessment and Imaging Biomarkers*,* (2) Exploring the Biology and Modulators of Breast Density*,* (3) Innovative Technology and Methods for Imaging Risk Assessment*,* (4) Frontiers in Breast Cancer Risk Assessment*,* and (5) Engaging Communities to Improve Risk Models and Screening Strategies.* In addition, the workshop included an innovative Breast Cancer Risk Board delivered in a tumor board-style format. Panelists included clinicians and advocates from Hawai‘i and Japan who presented real-world case studies, sparking a lively and collaborative discussion on individualized risk communication and management strategies. Here we summarize the presentations and discussion in each of the thematic sessions.

## Session summaries

***Session I: State of the Art in Risk Assessment and Imaging Biomarkers.*** *Moderator: John Shepherd*. The session opened with a personal perspective from **Joanne Hayashi**, President and Co-Founder of Breast Cancer Hawai’i and a breast cancer survivor, who shared her experience with breast cancer and the story behind building one of Hawai’i’s leading patient advocacy organizations. Her remarks grounded the scientific sessions that followed in the lived experience of the patients this research ultimately serves. Dr. **Gils** detailed long-term results from the DENSE trial, a randomized study assessing supplemental magnetic resonance Imaging (MRI) screening for women with extremely dense breasts. Her presentation emphasized the value of MRI in reducing interval cancers over three screening rounds, establishing a compelling case for tailored imaging strategies based on density. Dr. **Boddicker** explored the associations between cancer predisposition genes and breast density. His findings advanced understanding of how genetic risk intersects with imaging phenotypes, suggesting potential synergies in combined risk modeling. Dr. **van Grinsven** presented a multi-reader study evaluating the diagnostic accuracy of abbreviated breast MRI for women with dense breasts. The results demonstrated promising performance metrics for abbreviated protocols, offering a more efficient yet sensitive alternative to full MRI in high-risk screening. Dr. **Gierach** presented on the predictive value of mortality by mammographic breast density among women already diagnosed with breast cancer. Her work underscored the prognostic importance of breast density beyond initial risk assessment, highlighting opportunities for tailored surveillance and treatment planning. Dr. **Kerlikowske** contributed new data on the distribution of invasive and advanced breast cancer (stages 2 and above) risk stratified by density levels, reinforcing the critical role of density in identifying subpopulations most likely to benefit from intensified screening. Lastly, Dr. **Mahmoud** reported racial differences in background parenchymal enhancement (BPE), a novel imaging biomarker for breast cancer risk. His findings highlighted the necessity of imaging research across diverse populations to ensure biomarkers are valid in all women.

***Session II: Exploring the Biology and Modulators of Breast Density including Hormones***,*** Obesity***,*** and Environment.*** *Moderator: Kimberly Bertrand*. This session focused on the complex interplay between biological, hormonal, lifestyle, and environmental factors that influence breast density. Dr. **Park** opened the session with a plenary talk on insights drawn from Korea’s national mammographic screening program, highlighting shared risk between breast density and breast cancer and how increasing prevalence of dense breasts parallels increasing breast cancer incidence [8]. She also presented research showing a paradoxical lower risk of interval breast cancer associated with high breast density – attributed to higher prevalence of supplemental screening in this population [9] and how changes in breast density may influence risk [10]. Her work underscored the critical importance of data-driven screening strategies tailored to specific populations. Dr. **Terry** followed with a plenary presentation exploring how breast density research can serve as a lens to understand the influence of environmental exposures, including endocrine-disrupting chemicals, on global increases in breast cancer incidence, particularly among women < 50 years of age. Her presentation, drawing on data from family-based cohorts and other high-risk populations [11], emphasized the value of integrating environmental epidemiology with imaging biomarkers and of interrogating relevant windows of susceptibility, including critical developmental periods such as early life. In a proffered abstract talk, Dr. **Kehm** presented findings from a longitudinal study of adolescent girls, linking physical activity, body fat, and biomarkers of oxidative stress and inflammation to breast tissue composition [12], showing that breast tissue composition is influenced by both biological factors and modifiable external factors. These findings also highlight how these early-life exposures may shape long-term breast cancer risk. Dr. **Hurson’s**, proffered abstract talk focused on breast cancer survivors, in which she shared new findings on the impact of receipt of radiotherapy with or without endocrine therapy on changes in density of the contralateral breast. These results advance understanding of the etiology of contralateral breast cancer and also have important implications for risk-tailored surveillance.

***Session IV: Innovative Technology and Methods for Imaging Risk Assessment.*** *Moderator: Fredrik Strand*. This session highlighted emerging approaches that push imaging-based risk assessment beyond traditional descriptors and toward more actionable representations of individual breast cancer risk. The presentations collectively demonstrated how innovations in artificial intelligence, image-derived biomarkers, and workflow design may reshape early detection and prevention strategies.

The session opened with Dr. **Mainprize’s** presentation of Mammatus, a novel tool designed to quantify masking directly from mammograms. By measuring how breast tissue composition may obscure tumors, Mammatus offers a path to more tailored screening strategies, especially for women with dense breasts where missed cancers remain a pressing concern. This was followed by Dr. **Eriksson’s** work on a 10-year risk model that incorporates imaging features linked to modifiable risk factors such as breast density, weight, and benign breast disease. His findings suggest that image-derived phenotypes not only predict risk but may serve as surrogates for prevention targets, enabling dynamic and responsive risk assessments over time.

Building on the theme of integrated modelling, Dr. **Karssemeijer** presented an AI-based hybrid approach that combines lesion detection with mammographic texture analysis. By bringing together these complementary signals, the model demonstrated improved performance for both long-term risk estimation and triage applications, pointing toward systems that can simultaneously support individualized care and screening-program efficiency. The session concluded with Dr. **Gommers’** proposal to structure reading workflows according to breast density [13], placing more complex examinations earlier in the sequence. This operational refinement offered a reminder that innovation in screening need not be exclusively algorithmic; changes in workflow design can also enhance diagnostic accuracy and resource use. Together, the presentations illustrated a maturing field in which imaging contributes not only to finding cancers, but also to anticipating who develops them, understanding why some cancers are missed, and shaping screening pathways that are both equitable and effective.

**Session V: Frontiers in Breast Cancer Risk Assessment: Integrating Clinical**,** Omic**,** and Imaging Biomarkers.** *Moderator: Karla Kerlikowske*. This session delved into the integration of multi-dimensional data—clinical history, imaging features, and molecular biomarkers, to enhance breast cancer risk prediction and address long-standing challenges in precision screening. The session began with three abstract presentations highlighting the transformative role of artificial intelligence (AI) in longitudinal mammography analysis. **Dr. Hernandez** demonstrated how AI models applied to sequential mammograms can improve long-term risk prediction, while Dr. **Jiang** presented a validated dynamic model that uses prior mammograms to estimate 5-year breast cancer risk. Dr. **Kaul** compared traditional clinical models to AI-based mammography models, showing that image-derived risk tools can surpass standard approaches, particularly in intermediate-risk populations. Dr. **Shen** offered a forward-looking view of deep learning applied to multimodal and longitudinal imaging data. His work illustrated how AI models trained on diverse inputs, from imaging to time-series data, can capture nuanced temporal and anatomical patterns predictive of future cancer. Dr. **Arasu** presented a comparative analysis of 15-year risk models including AI, clinical, and polygenic scores, showing notable differences in predictive power and calibration. Dr. **Sieh** examined the performance of genomic and radiomic models across racial/ethnic subgroups, highlighting the need for equity in training datasets. Finally, Ms. **Bunnell** discussed a matched case-control study in an ethnically diverse cohort, emphasizing how incorporating radiomic and AI features can improve risk prediction in populations historically underrepresented in model development. This session underscored the value and necessity of integrating multi-source data for precise, equitable breast cancer risk assessment. The talks highlighted how complexity in both patient data and population diversity can be addressed through thoughtful model design and validation.

***Session VI: Engaging Communities to Improve Risk Models and Screening Strategies Toward Health Quality for All Women.*** *Moderator: Celine Vachon.* This session emphasized the critical role of community engagement, health equity, and cultural sensitivity in refining risk prediction and screening strategies for breast cancer, particularly among diverse and underserved populations. Dr. **Minck**, a breast cancer patient advocate, presented work on effective communication of breast density and cancer risk. Drawing on global insights and consumer feedback, she stressed the importance of centering patient voices to improve understanding, trust, and participation in breast health strategies. Dr. **Hubbard** followed with a plenary talk highlighting the use of synthetic data to explore disparities in mammography performance and risk-based surveillance recommendations. Her work underscored the value of simulation methods to identify potential biases and optimize screening guidelines for equitable outcomes. Dr. **Nickel** presented findings from a randomized controlled trial embedded in Australia’s national screening program. Her talk revealed the psychosocial and service impacts of breast density notification, emphasizing the balance between patient empowerment and unintended anxiety or confusion. Dr. **McCarthy** addressed how breast density and race contribute to disparities in breast cancer detection and outcomes. Her plenary talk reinforced the need for inclusive data and models that reflect population-level variation. Lastly, Dr. **Strand** concluded the session with results from a multi-region study on fairness in AI-based mammography screening. His analysis of the performance of three AI-detections across geographic and demographic settings revealed persistent disparities and the urgent need for fairness-aware design in medical AI. Three of the talks (Hubbard, McCarthy and Strand) highlighted the importance of robust AI based models and breast density measures across race and ethnicity. First, the estimation of breast density itself can lead to biases in downstream recommendations for supplemental screening. Much of the legislation to date involves additional screening measures for women with dense breasts, defined as Breast Imaging Reporting and Data System (BI-RADS) C or D. However, we know that the clinical BI-RADS measure as well as some commercially available percent measures result in inaccurate breast density assessments, due to its confounding with body mass index. Dr. Mc Carthy emphasized the differences in distributions of density measures particularly in Asian and Black women, when not taking into account BMI. The implications of less accurate density measures will result in reduced supplemental screening recommendations to some (Black women) and more than necessary to others (Asian women). A solution would be to make recommendations instead, using an age and adjusted density measure, as recommended by Dr. John Hopper, or use the more robust dense volume measure, an absolute measure of dense tissue in the breast, which shows less variability by BMI or race and ethnicity. These measures may better target supplemental screening recommendations and should be explored.

Secondly, Dr. Strand’s findings of differential AI detection model scores by race and ethnicity, estimated from mammograms from women with breast cancer case further emphasized the importance of robust and translational mammographic measures. Although all women had cancer, mammograms up to 4 years prior to cancer showed that Asian women had statistically significantly lower AI detection scores than Swedish women. Importantly, adjustment for known differences in breast density did not correct these differences. These data were extremely enlightening as many AI detection models being used in practice today do not consider racial and ethnic differences. Inequities or bias is created by training these AI models in populations of primarily European ancestries. Solutions could be to train and validate in multiple races and ethnicities, improve calibration within ethnicity, or use other statistical methods to fine-tune models using race and ethnic information.

Dr. Hubbard’s talk reinforced that data availability via the electronic health record (HER) can create bias which will translate to risk models and surveillance, if not considered. Availability, type and amount of data will vary by clinical practice, access, patient demographics, with historically underserved groups having less comprehensive and poor quality data. Differential outcome ascertainment also can exacerbate algorithm fairness. Investigations to understand and quantify this bias will be necessary, in particular in the AI setting, where all data is generally considered equal. With novel AI risk models being developed for breast cancer detection and risk prediction, the right study designs will need to consider data availability, race and ancestry, outcome ascertainment and account for error in model development and evaluation. Taken together, this session advanced the conversation from algorithmic innovation to real-world application, showing that equitable cancer risk assessment requires as much attention to community context and communication as to computational power.

### Breast cancer risk board

***An Open Discussion of Cases in the Style of a Tumor Board.*** *Moderator: Fredrik Strand*. This session introduced an innovative format to the IBDW: a risk board modeled on the clinical tumor board but oriented entirely toward the pre-diagnosis setting. Where traditional tumor boards focus on treatment planning for patients with confirmed cancer, this session centered on clinical dilemmas in screening, prevention, and risk management for individuals at elevated risk. Cases were developed by Dr. **Hideko Yamauchi** (Breast Surgical Oncologist, UHCC), with input from the full panel, and drawn from real-world clinical experience in Hawai’i and Japan. The panel brought together a deliberately cross-disciplinary and cross-cultural group: Dr. **Hideko Yamauchi** (Surgeon, UHCC), Dr. **Teruo Yamauchi** (Oncologist, UHCC), Dr. **Masako Kataoka** (Radiologist, Kyoto University Hospital), and **Joanne Hayashi** (patient advocate, Breast Cancer Hawai’i), with additional contributions from Drs. Strand, Kerlikowske, Shepherd, and **Jami Fukui** (UHCC). Each case was presented at key decision points, with the audience polled in real time using Mentimeter before panelists revealed how the clinical team had proceeded. Cases explored questions at the frontier of individualized risk management, including screening frequency, selection of imaging modalities and genetic testing, chemoprevention, and how patient characteristics such as age, breast density, family history, and cultural context shape clinical decisions. Dr. Kataoka’s Japanese clinical perspective added meaningful international dimension, highlighting differences in risk communication and supplemental screening practices across health systems. The Risk Board was among the most enthusiastically received sessions of the workshop, and its format is being considered as a recurring element of future IBDW meetings.

## Key gaps and emerging opportunities

Several important gaps and opportunities emerged across the workshop sessions. A central unresolved question is whether AI imaging models alone can match the predictive power of models that integrate both imaging and clinical risk factors, with implications for how next-generation tools are designed and validated. Standardization of density measures and AI model calibration across diverse populations remains an urgent priority, as does investment in the data infrastructure needed to support interoperability across registries and health systems. Dr. Terry identified critical gaps in understanding how environmental exposures, particularly endocrine-disrupting chemicals, influence breast cancer risk, emphasizing the value of studying breast density as a proximal surrogate marker and the need for research focused on early-life windows of susceptibility, including the in utero period, early childhood, and adolescence. Dr. Kehm echoed this gap, underscoring the need for longitudinal studies to determine how adolescent breast tissue composition shapes long-term cancer risk. On the opportunity side, non-imaging approaches such as optical spectroscopy offer promising low-cost alternatives to MRI and dual-energy X-ray Absorptiometry (DXA) for measuring breast tissue composition in young populations. Additional emerging opportunities include the use of harmonized databases, cross-disciplinary collaborations linking imaging with omic and environmental data, and risk-tailored surveillance strategies for breast cancer survivors, an area where Dr. Hurson’s findings revealed a striking paucity of evidence on contralateral breast cancer risk following treatment.

## Panel discussions and future directions

Several speakers acknowledged the critical importance of inclusive research, acknowledging known racial disparities in mammography performance, accurate risk prediction, and breast cancer outcomes and how differences in breast density across populations may contribute. There is great potential in applications of AI to improve breast cancer outcomes, but validation of these tools in multiple diverse populations is critically necessary for equitable roll-out.

Regarding recommendations for policy, funding, and research collaboration, there is increasing need for a global perspective in breast cancer research. Most countries are experiencing an increase in incidence, with a steep rise among women under age 50. Understanding drivers of this increase requires a focus on early life risk factors, including breast tissue composition. Consideration of population-specific incidence patterns may also reveal insights. For example, as Dr. Park noted, breast cancer incidence in Korea is rising faster than in any other country and, like many Asian countries, displays a unique age-incidence pattern with peak incidence around age 50; this trend parallels increases in the prevalence of dense breasts. Dr. Terry pointed out the value of research conducted in high-susceptibility populations, where signals may be stronger in the setting of chronic low-level exposures and where evidence for associations of environmental exposures and breast density and cancer risk are more consistent [14, 15].

## Summary and call to action

The 11th IBDW demonstrated that the field of breast cancer risk assessment is at an inflection point, with AI-based imaging biomarkers maturing rapidly, biological and environmental determinants of breast density coming into sharper focus, and the imperative for equity in risk prediction and screening now firmly established as a scientific priority, not merely an aspiration. Across sessions, a clear consensus emerged that progress will require standardization of density measures, rigorous validation of AI models across racially and ethnically diverse populations, integration of multi-source clinical and omic data, and sustained investment in community engagement and patient-centered communication. Researchers, clinicians, funders, and policymakers are urged to prioritize these goals collaboratively and with urgency, recognizing that inequities in model development and screening access risk widening, rather than narrowing, existing disparities in breast cancer outcomes. The 12th IBDW will be held in Maui, Hawai’i on June 2–4, 2027, where the field will reconvene to report progress on these priorities and chart the next steps forward.

## Data Availability

Not Applicable.

## Abbreviations

AI: Artificial intelligence
BI: RADS–breast imaging reporting and data system
BPE: Background parenchymal enhancement
CT: Computed tomography
DXA: Dual–energy X–ray absorptiometry
EHR: Electronic health record
IBDW: International breast density workshop
MRI: Magnetic resonance imaging
NCI: National cancer institute
US: United States

## References

[1] Boyd NF, Guo H, Martin LJ, Sun L, Stone J, Fishell E, Jong RA, Hislop G, Chiarelli A, Minkin S. Mammographic density and the risk and detection of breast cancer. N Engl J Med. 2007;356(3):227–36. 17229950 10.1056/NEJMoa062790

[2] McCormack VA, dos Santos Silva I. Breast density and parenchymal patterns as markers of breast cancer risk: a meta-analysis. Cancer Epidemiol Biomarkers Prev. 2006;15(6):1159–69. 16775176 10.1158/1055-9965.EPI-06-0034

[3] Lange JM, Gard CC, O’Meara ES, Miglioretti DL, Etzioni R. Breast density and risk of breast cancer: masking and detection bias. Am J Epidemiol. 2025;194(2):441–8. 39098823 10.1093/aje/kwae245 PMC11815494

[4] Liao JM, Lee CI, editors. Strategies for mitigating consequences of federal breast density notifications. JAMA Health Forum; 2023: American Medical Association. 10.1001/jamahealthforum.2023.2801 37682552

[5] Kressin NR, Slanetz PJ, Gunn CM. Ensuring clarity and understandability of the FDA’s breast density notifications. JAMA. 2023;329(2):121–2. 36508205 10.1001/jama.2022.22753 PMC10152312

[6] Seely JM, Peddle SE, Yang H, Chiarelli AM, McCallum M, Narasimhan G, Zakaria D, Earle CC, Fung S, Bryant H. Breast density and risk of interval cancers: the effect of annual versus biennial screening mammography policies in Canada. Can Assoc Radiol J. 2022;73(1):90–100. 34279132 10.1177/08465371211027958

[7] Stone J, Marriott R, Orellana M, Lee E, Porter G. The Impact of Breast Density Notification on Interval Cancer Rates. J Med Imaging Radiat Oncol. 2026. 10.1111/1754-9485.70067 41562507

[8] Kim S, Tran TXM, Park B. Trends in breast density and other risk factors for breast cancer and associations with trends in the incidence of breast cancer in Korean women. Maturitas. 2024;189:108070. 39173537 10.1016/j.maturitas.2024.108070

[9] Song H, Tran TXM, Kim S, Park B. Risk factors and mortality among women with interval breast cancer vs screen-detected breast cancer. JAMA Netw Open. 2024;7(5):e2411927. 38767918 10.1001/jamanetworkopen.2024.11927 PMC11107304

[10] Park B, Chang Y, Ryu S, Tran TXM. Trajectories of breast density change over time and subsequent breast cancer risk: longitudinal study. BMJ. 2024;387. 10.1136/bmj-2024-079575 PMC11684031 39797631

[11] Zeinomar N, Oskar S, Kehm RD, Sahebzeda S, Terry MB. Environmental exposures and breast cancer risk in the context of underlying susceptibility: a systematic review of the epidemiological literature. Environ Res. 2020;187:109346. 32445942 10.1016/j.envres.2020.109346 PMC7314105

[12] Kehm RD, Lilge L, Walter EJ, Santella RM, White ML, Herbstman JB, Perera F, Miller RL, Terry MB. Recreational physical activity and biomarkers of breast cancer risk in a cohort of adolescent girls. Breast Cancer Res. 2026. 10.1186/s13058-025-02216-1 PMC12870528 41501828

[13] Gommers JJ, Verboom SD, Duvivier KM, van Rooden J-K, van Raamt AF, Houwers JB, Naafs DB, Duijm LE, Abbey CK, Webster MA. Enhancing radiologist reading performance by ordering screening mammograms based on characteristics that promote visual adaptation. Radiology. 2024;313(1):e240237. 39377678 10.1148/radiol.240237 PMC11535868

[14] Houghton LC, Lui ML, Terry MB. A life course approach to cancer. A Life Course Approach to the Epidemiology of Chronic Diseases and Ageing. 2025:121.

[15] Kehm RD, Lloyd SE, Burke KR, Terry MB. Advancing environmental epidemiologic methods to confront the cancer burden. Am J Epidemiol. 2025;194(1):195–207. 39030715 10.1093/aje/kwae175 PMC11735972

